# *PGC-1α* Methylation, miR-23a, and miR-30e Expression as Biomarkers for Exercise- and Diet-Induced Mitochondrial Biogenesis in Capillary Blood from Healthy Individuals: A Single-Arm Intervention

**DOI:** 10.3390/sports10050073

**Published:** 2022-05-06

**Authors:** Ulrike D. B. Krammer, Alexandra Sommer, Sylvia Tschida, Anna Mayer, Stephanie V. Lilja, Olivier J. Switzeny, Berit Hippe, Petra Rust, Alexander G. Haslberger

**Affiliations:** 1 Department of Nutritional Science, University of Vienna, A-1090 Vienna, Austria; uk@healthbiocare.at (U.D.B.K.); sylvia.tschida@hotmail.com (S.T.); anna_mayer.1@gmx.at (A.M.); stephanie.lilja@univie.ac.at (S.V.L.); bh@healthbiocare.at (B.H.); petra.rust@univie.ac.at (P.R.); 2 HealthBioCare GmbH, A-1090 Vienna, Austria; switzeny@healthbiocare.at; 3 Center for Molecular Biology, University of Vienna, A-1030 Vienna, Austria; alexandra-sommer95@gmx.de

**Keywords:** mitochondrial biogenesis, epigenetic, PGC-1α, polyphenols, concurrent training

## Abstract

Healthy mitochondria and their epigenetic control are essential to maintaining health, extending life expectancy, and improving cardiovascular performance. Strategies to maintain functional mitochondria during aging include training; cardiovascular exercise has been suggested as the best method, but strength training has also been identified as essential to health and healthy aging. We therefore investigated the effects of concurrent exercise training and dietary habits on epigenetic mechanisms involved in mitochondrial (mt) functions and biogenesis. We analyzed epigenetic biomarkers that directly target the key regulator of mitochondrial biogenesis, *PGC-1α*, and mtDNA content. Thirty-six healthy, sedentary participants completed a 12-week concurrent training program. Before and after the intervention, dried blood spot samples and data on eating habits, lifestyle, and body composition were collected. MiR-23a, miR-30e expression, and mtDNA content were analyzed using real-time quantitative polymerase chain reaction (qPCR) analysis. *PGC-1α* methylation was analyzed using bisulfite pyrosequencing. MiR-23a, miR-30e expression, and *PGC-1α* methylation decreased after the intervention (*p* < 0.05). *PGC-1α* methylation increased with the consumption of red and processed meat, and mtDNA content increased with the ingestion of cruciferous vegetables (*p* < 0.05). Our results indicate that concurrent training could improve mitochondrial biogenesis and functions by altering the epigenetic regulation. These alterations can also be detected outside of the skeletal muscle and could potentially affect athletic performance.

## 1. Introduction

**Figure 1:**
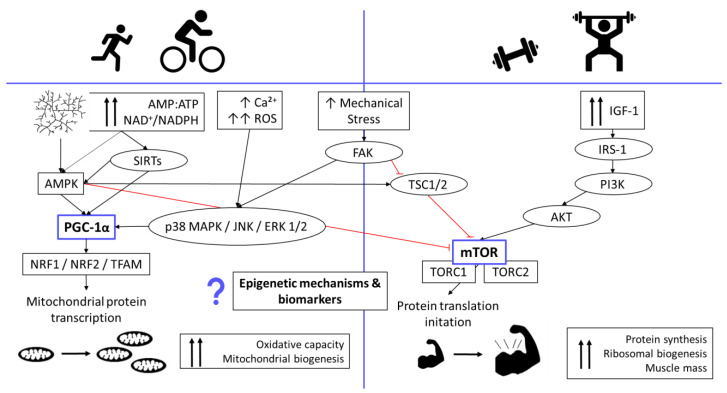
A graphic representation of the main signaling pathways in the regulation of skeletal muscle hypertrophy and mitochondrial biogenesis. Voluntary training alters primary signals such as mechanical stress or muscle energy status (AMP:ATP Ratio) and activates kinases/phosphatases in order to convey a specific exercise-induced signal. These pathways are regulated at several points, with significant crosstalk among the numerous signaling pathways creating a highly sensitive and complex transduction network. (The illustration was inspired by Methenitis [1] and Hawley et al. [11]). AMPK = AMP-activated Protein Kinase, PGC1-α = PPAR-γ Coactivator-1α, NRF1= Nuclear Respiratory Factor 1, NRF2 = Nuclear Factor Erythroid 2-related Factor 2, TFAM = mitochondrial Transcription Factor A, FAK = Focal Adhesion Kinase, TSC = Tuberous Sclerosis Complex, AKT = Protein Kinase B, mTOR = mammalian Target Of Rapamycin, IGF-1 = Insulin-like Growth Factor 1, PI3K = Phosphatidylinositide 3-Kinases, p38 MAPK = p38 Mitogen-activated Protein Kinase, JNK = Jun amino-terminal Kinase, ERK 1/2 = Extracellular signal-regulated Kinases 1/2, IRS-1 = Insulin Receptor Substrate 1, SIRT = Sirtuin, TORC1/2 = mTOR Complex 1/2, ROS = Reactive Oxygen Species, AMP:ATP = Adenosine Monophsphate: Adenosine Triphosphate ratio, NAD = Nicotinamide Adenine Dinucleotide, NADP = Nicotinamide Adenine Dinucleotide Phosphate.

In addition to maintaining or improving general health, exercise training aims to improve an individual’s physical and conditioning performance by using physical loads as a potential stimulus to induce muscle growth in the human body. These specific reactions or training-related adaptions, triggered by endurance or resistance training, occur via a complex network of several signaling pathways (Figure 1) [1,2].

Endurance training (Figure 1, left side) induces signaling pathways such as peroxisome proliferator-activated receptor γ coactivator 1α (PGC-1α), p38 mitogen-activated protein kinase (p38 MAPK), and AMP-activated protein kinase (AMPK), all of which underlie the cellular processes that stimulate mitochondrial biogenesis and angiogenesis. This enables metabolic adaptions that lead to improvement in endurance performance [1,2]. Mitochondrial biogenesis is defined as how existing mitochondria grow and divide to create new mitochondria. This takes place on the one hand by cell division, but also in response to an oxidative stimulus by increasing the cell’s energy requirements, by electrical stimulation, by hormones, in certain mitochondrial diseases, and by physical training [3,4]. Repeated training sessions lead to an improvement in mitochondrial volume and function, thereby improving oxidative capacity. Reactive oxygen species (ROS) or mitochondrial stress occur, and low ROS levels or mild mitochondrial stress are known to induce adaptive responses that affect both short-term (e.g., metabolic) and long-term (e.g., longevity) health—responses that are referred to as mitohormesis [5,6]. Healthy physiology of the mitochondria is essential for health and longevity, whereby dysfunction is related to the pathogenesis of various diseases, such as type 2 diabetes and cardiovascular diseases [7,8]. PGC-1α promotes mitochondrial biogenesis by activating various transcription factors, e.g., nuclear respiratory factor 1 (NRF1) and nuclear factor erythroid 2–related factor 2 (NRF2), which promote the expression of mitochondrial transcription factor A (TFAM). TFAM increases the transcription of critical mitochondrial enzymes, which drives mtDNA replication [3]. Furthermore, PGC-1α plays a major role in regulating cellular energy metabolism and in muscle fiber type determination (e.g., formation of slow-twitch muscle fibers), stimulates mitochondrial biogenesis, and has a regulatory function in lipid metabolism. Therefore it is very likely that PGC-1α is also involved in diseases such as obesity and diabetes [9,10].

On the other side, resistance training (Figure 1, right side) activates the molecular pathway responsible for muscle hypertrophy and stimulates the myofibrillar protein synthesis via insulin-like growth factor 1 (IGF-1) and the protein kinase B (AKT)-mammalian target of the rapamycin (mTOR) pathway, which leads to an increase in muscle mass and protein synthesis. In addition, it is assumed that high-volume, moderate, repeated, or frequent endurance training can negatively influence the adaptions caused by resistance training, presumably by inhibiting the activation of the AKT-mTOR-signaling pathway through AMPK [1,2]. In contrast, resistance exercise can stimulate mitochondrial biogenesis in the skeletal muscles, possibly via focal adhesion kinase (FAK) [1,12].

Training also leads to transcriptional, translational, and post-translational changes. Even if these molecular mechanisms are not yet fully understood, it is assumed that epigenetic mechanisms play a role in training-related adaptions [13]. Through epigenetic mechanisms, cells can react to various environmental stimuli, adapt gene expression to new circumstances, and regulate mitochondrial functions. Epigenetic mechanisms include DNA modifications (i.e., methylation), the regulation of gene expression by non-coding RNAs (e.g., miRNAs), and post-translational modifications of histones [14]. It has already been described that, after a strenuous training session, the promoter regions of genes that are important for training metabolism (e.g., *PGC-1α*) are hypomethylated, with a simultaneous increase in the mRNA level. Furthermore, it has been shown that the expression of some miRNAs differs after exercise, which could affect skeletal muscle regeneration, gene transcription, and mitochondrial biogenesis [13,15]. Often-discussed examples are miR-23, miR-696, and miR-30e, which have *PGC-1α* as a predicted target [16,17,18]. Endurance training seems to downregulate miR-23a expression, which promotes mitochondrial biogenesis by the upregulation of *PGC-1α*. In mice, it is also reported that overexpression of miR-23a downregulates the protein abundance of PGC-1α in skeletal muscles. However, the exact role and function of miR-23a in the mitochondria is currently unknown, but miR-23a appears to inhibit myogenic differentiation and consequently regulates the contractile function of mature myofibers [16,17]. Also, miR-30e seems to be involved in myogenic differentiation. In vivo studies have shown that overexpression of miR-30e reduces *PGC-1α* expression and increases the formation of glycolytic myotubes [19].

The main purpose of this work was to investigate whether a medium-term sports intervention focusing on endurance training and resistance training causes long-term epigenetic modifications which, according to the literature, have an influence on mitochondrial biogenesis or play a role in the metabolic and signaling pathways described above (Figure 1). Since endurance and resistance training at the same time are believed to have more beneficial effects on general health, mitochondrial biogenesis, disease prevention, and healthy aging than each modality alone [20,21], we considered the effects of endurance and resistance training. Furthermore, we hypothesized that epigenetic biomarkers in the blood (e.g., miRNAs, CpG methylations) reflect processes or changes in the muscles and the general state of health, as components from the skeletal muscles are released into the systemic circulation via extracellular vesicles [22]. However, it is not only physical activity that can influence health, physical performance, and mitochondrial biogenesis; nutrients, e.g., bioactive food components like phytochemicals, or diets such as fasting or caloric restriction [23,24] can do this as well. For example, the intake of phytochemicals, as in exercise or fasting, leads to the activation of AMKP and SIRT1 and thus ultimately influences mitochondrial biogenesis (Figure 1) [25,26]. Furthermore, there is an association between body composition and mitochondrial energy metabolism. Mitochondria regulate maximum oxygen consumption and control several important functions in adipose tissue. Total body fat is also correlated with impaired mitochondrial function [27,28]. Therefore, we additionally investigated whether dietary habits or body composition influence health or the selected biomarkers.

## 2. Materials and Methods

### 2.1. Subjects and Experimental Design

This study includes the data of 36 healthy, sedentary women and men, who completed a 12-week concurrent training intervention, including 60 min of endurance training twice a week and total-body resistance training three times a week (Table 1). The subjects were on average 31.86 ± 8.02 years old. The mean body mass index (BMI) was 24.17 ± 2.95 kg/m^2^, and over 58% of the participants had a BMI in the normal range (18.5–24.9 kg/m^2^). Twenty-one participants were female (58.3%) and fifteen participants were male (41.7%). Exclusion criteria were elite athletes, individuals who have participated extensively (for the past 2 years) in competitive sports, and individuals with a history of chronic illness or medication use. All participants were advised to maintain their lifestyle and dietary habits throughout the intervention. Before [T0] and after [T1] the intervention, food frequency questionnaires, health records, dried blood spots (DBS), and body composition data (e.g., lean body mass (LBM), body fat mass (BFM), using bioelectrical impedance analysis (BIA, Nutriguard-MS)) were collected and basal metabolic rate (the number of calories the body needs for basic life-support functions) calculated. Participants were instructed to report for sample collection and body composition measurement between 24 and 48 h after their last training session. In addition, it was precisely noted when the respective participants came to their appointment after the last training session. Most participants (75%) came within 32 h after their last training session. Only nine participants (25%) came between 32 and 48 h after their last training. This was also considered in the statistical evaluation of the data and considered separately. In accordance with the declaration of the Viennese Human Ethics Committee, all the human subjects gave their written approval for the use of the data.

### 2.2. Training Plan

Strength Training: In the first two to four weeks the participants were accustomed to the equipment/strength exercises with no or little additional weight, so as to learn the correct execution. After the “familiarization phase”, the training was carried out with additional weight, which was increased by 1.25 to 5 kg (depending on the exercise machine) as soon as the participants managed 3 sets with 12 repetitions. The training alternated between plans A and B so that there was a break of approximately 48 h between the different strength exercises (Table 1).

Endurance Training: The endurance training units (treadmill or ergometer) should initially be carried out for 30 min at an intensity of 60–85% maximum heart rate. After an adjustment period of three to four weeks, the duration was extended by 5 to 10 min per week over the next weeks, up to a duration of at least 60 min in the last three to four weeks (Table 1).

### 2.3. Dried Blood Spot Sample Preparation (RNA and DNA Extraction)

The total RNA was isolated from the dried blood spots using the *miRNeasy Micro Kit* (Qiagen, Hilden, Germany) and the total DNA using the *QIAamp^®^ DNA Mini Kit* (Qiagen, Hilden, Germany), according to the manufacturer’s protocol. Quantity and quality were assessed using a *NanoDropTM One/OneC Microvolume UV-Vis Spectrophotometer* (ThermoFisher Scientific, Waltham, MA, USA), and the samples were then stored at −20 °C.

### 2.4. Bisulfite Conversion and Pyrosequencing

The gDNA samples were bisulfite-converted using the *EpiTect Bisulfite Kit* (Qiagen, Hilden, Germany) according to the manufacturer’s protocol. The bisulfite-converted DNA (bisDNA) samples were diluted to 20 ng/µL. The polymerase chain reaction (PCR) of the diluted bisDNA samples was performed with the *PyroMark^®^ Kit* (Qiagen, Hilden, Germany). According to our established protocol, there was an initial activation period of 15 min. at 95 °C, a 3-step cycle of denaturation (94 °C for 30 s), annealing (58 °C for 5 s), and extension (72 °C for 15 s) for 45 cycles. The PCR process was terminated with a final extension period of 72 °C for 10 min. PyroMark assays (biomers.net, Ulm, Germany), primer sequences, and the sequence to analyze, based on the work by Hunter et al. 2019 [15], are listed in Table 2.

Gel electrophoresis was performed on a 2% agarose gel using *GeneRuler 100 bp DNA Ladder* (ThermoFisher Scientific, Waltham, MA, USA) to check the PCR products. CpG methylation was determined using *PyroMark Q24* (Qiagen, Hilden, Germany) and *PyroMark Gold Q24 Reagents* (Qiagen, Hilden, Germany). The nucleotide dispensing order was generated by entering the sequence to be analyzed into the *PyroMark Q24 software version 2.0.8* (Qiagen, Hilden, Germany). The methylation at each CpG site was evaluated using the *PyroMark Q24 software* in the CpG mode.

### 2.5. Real-Time Quantitaive PCR (qPCR)

The mtDNA content was measured in the gDNA samples. For the qPCR, a *StepOne Plus real-time PCR Detection System* (Applied Biosystems, Waltham, MA, USA), a single-copy gene primer (fw: GCT TCT GAC ACA ACT GTG TTC ACT AGC, rev: CAC CAA CTT CAT CCA CGT), mtDNA primer (fw: CAT CTG GTT CCT ACT TCA GGG, rev: TGA GTG GTT AAT AGG GTG ATA GA; Biomers, Ulm, Germany) [29] and a *LightCycler^®^ 480 Sybr^®^ Green I Master Mix* (Roche, Munich, Germany) were used. For the miRNA analyses, we used *the TaqMan™ Advanced microRNA cDNA Synthesis Kit* and *TaqMan™ Advanced microRNA assays* (miR-23a-3p, -30e-3p, and endogenous controls miR-24-3p and -93-5p) under the default settings on *QuantStudio™ 3* from ThermoFisher Scientific, Waltham, MA, USA. Fold changes were calculated with the formula 2^−∆∆Ct^ [30].

$$\Delta Ct=Ct^{target}-Ct^{endogenous\;control}\;\Delta \Delta Ct=\Delta Ct^{T1}-\Delta Ct^{T0}$$

### 2.6. Statistical Analysis

The statistical analysis was carried out using IBM SPSS Statistics 20 and GraphPad Prism 6. All data are represented as mean ± standard deviation (SD). Paired t-tests were used to analyze the different time points for parametric and Wilcoxon tests for nonparametric values. Correlations between methylation, miRNA expression, and body composition were tested using Pearson correlation and linear regression. Correlations between dietary habits, methylation, and mtDNA were tested using Spearman’s Rho correlation and Kendall’s tau. A *p*-value less than or equal to 0.05 was defined as significant for all tests.

## 3. Results

### 3.1. Molecular and Physiological Changes Following 12 Weeks of Concurrent Training

Body weight (kg, *p* = 0.009), BMI (kg/m^2^, *p* = 0.009), basal metabolic rate (kcal, *p* = 0.003), LBM (kg, *p* = 0.017), and BFM (kg, *p* = 0.000) or body fat percentage (BFP; %, *p* = 0.000) improved significantly through the 12-week concurrent training. Body weight, BMI, and BFM could be reduced, and basal metabolic rate and LBM increased (Table 3).

**Figure 2:**
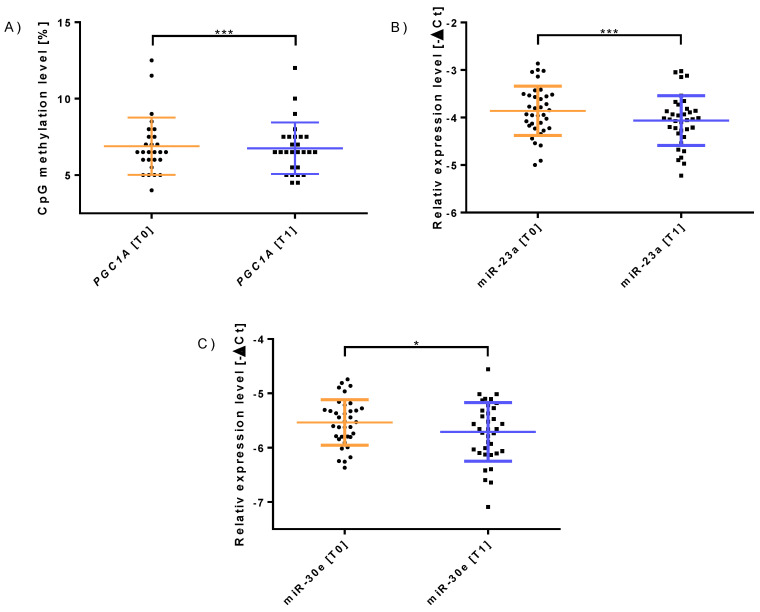
Molecular changes following 12 weeks of concurrent training. (**A**) Change in *PGC-1α* methylation during the intervention (*n* = 27). (**B**) Change in miR-23a expression during the intervention (*n* = 36). (**C**) Change in miR-30e expression during the intervention (*n* = 36). The results are expressed as mean ± SD. * Shows significant *p*-values below 0.05 and *** *p*-values below 0.03 (paired *t*-test).

Furthermore, the 12-week intervention changed the miR-23a (*n* = 36, *p* = 0.023, 0.92-fold, Figure 2B) and miR-30e (*n* = 36, *p* = 0.047, 0.94-fold, Figure 2C) expression significantly. Participants whose capillary blood samples were collected 24 to 32 h after their last training session showed a change in *PGC-1α* methylation (*n* = 27, *p* = 0.024, Figure 2A) and expression of miR-23a (*n* = 27, *p* = 0.028, Table 4). Furthermore, there were no differences in *PGC-1α* methylation, mtDNA, miR-23a, and -30e expression between sex, smoker (*n* = 11, 30.6%) or non-smoker (*n* = 25, 69.4%) status, or age, so we included the entire study population for further analysis.

### 3.2. Correlations between Eating and Lifestyle Habits and Anthropometric Data with Selected Mitochondria- and Exercise-Related Markers Independent of the Intervention

**Figure 3:**
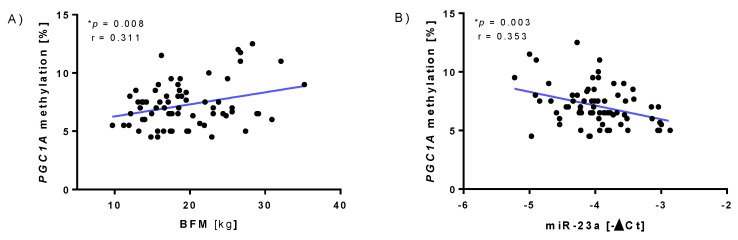
Correlations, independent of the intervention. (**A**) Linear regression between body fat mass (BFM, kg) and *PGC1A* methylation. (**B**) Linear regression between *PGC1A* methylation and miR-23a expression. * Shows significant *p*-values.

A positive correlation between *PGC-1α* methylation and body fat mass (BFM, kg) was obtained (*p* = 0.008, Figure 3A). As described in the literature, increasing *PGC-1α* methylation negatively correlated with miR-23a expression (*p* = 0.003, Figure 3B).

The participants who reported consuming red or processed meat at least 4 times a week (above the limit of the recommendation) or daily had higher levels of *PGC-1α* methylation (mean methylation 8.292 ± 2.147, *p* = 0.022) than those who reported consuming less or no red or processed meat (mean methylation 6.758 ± 1.686). Also, intake of cruciferous vegetables (e.g., broccoli, cabbage, brussels sprouts) and mtDNA content (*p* = 0.043) correlated positively (see Table 3 for intake frequencies). Participants who reported regularly, at least once a week, consuming cruciferous vegetables had higher mtDNA content (mean mtDNA 6.095 ± 0.408) than those who reported rarely or never consuming cruciferous vegetables (mean mtDNA 5.868 ± 0.327).

## 4. Discussion

The main aim of this study was to examine whether a medium-term concurrent exercise intervention induces measurable epigenetic changes in dried capillary blood samples (DBS), which allow conclusions to be drawn about a person’s fitness or health and would be suitable as biomarkers for exercise- and diet-induced mitochondrial biogenesis. Additionally, we analyzed the influence of lifestyle and eating habits on these biomarkers and the amount of mitochondrial DNA (mtDNA).

Chronic sustained concurrent exercise training decreased *PGC-1α* methylation levels and led to a downregulation of miR-23a and -30e in the capillary blood samples (DBS) for up to 32 h after a training session. These results are confirmed by the literature, since downregulation of miR-23a leads to an upregulation of *PGC-1α*, promoting mitochondrial biogenesis and improving endurance performance [16]. After more than 32 h, however, we could no longer detect a significant change in the methylation of *PGC-1α*. This is in accordance with the results of Barrès et al. [31], who also discovered no difference in methylation 48 h after a three-week training program. This suggests that hypomethylation is a transient mechanism that needs to be repeatedly stimulated, underlining the importance of regular exercise to benefit from the positive effects of *PGC-1α* expression, e.g., activation of mitochondrial biogenesis with subsequent improvement in endurance performance.

Furthermore, it is described that exercise-related changes in DNA methylation and miRNA expression patterns can be detected in skeletal muscles [32] and in leukocytes or neutrophils [15,33]. The currently available literature does not provide information on how long these changes last or can be detected after exercise, as explanatory studies are not yet available, and samples are usually taken directly or a few hours after exercise. But during exercise and partly during recovery periods, components such as mitochondria, metabolites, and muscle-enriched miRNAs (myomiRs) are “flushed” from the skeletal muscles into the systemic circulation [22,34]. This could explain why we observed changes in miR-23a and -30e expression but not in *PGC-1α* methylation in our DBS samples more than 32 h after the intervention. Like miR-23a, miR-30e is also involved in myogenic differentiation or muscle fiber formation, which presumably takes place in the regeneration phase [17,19]. It can be assumed that downregulation of miR-30e favors the formation of type 1 muscle fibers, which are particularly advantageous for long-term endurance units, as overexpression increases the formation of glycolytic or type 2 muscle fibers, so exercise possibly has an impact on type of muscle fiber formation [19,35]. Therefore, our results suggest that endurance capacity is improved by concurrent exercise training and by downregulating miR-30e and can possibly influence mitochondrial biogenesis. This is further reinforced by the correlation between the methylation of *PGC-1α* and the miR-23a expression. We suspect that there are two “fronts” through which personal athletic performance can be influenced, either through miR-23a or *PGC-1α* alone or through a synergistic effect, or both. This indicates that individuals respond differently to the different regulation systems.

Mitochondria are interconnected and highly mobile cell organelles that undergo dynamic changes in terms of physiological and pathological modifications. During the life of a cell, there is a relatively stable equilibrium in a multitude of processes: fission, fusion, and biogenesis [36,37]. Maintaining this balance, and the fact that mitochondria can fuse with each other, causing the mtDNA to mix, could be a possible explanation for why we did not see any change in the mtDNA content. As mentioned, the timing of sampling and the intensity (effort and duration) of the training seem to be of great concern, including how many mitochondria are released from the muscle into the bloodstream to detect changes of mtDNA [38]. Further studies as well as a comparison between muscle biopsy samples and DBS could clarify this.

However, we discovered an association between body fat mass (BFM) and *PGC-1α* methylation. The participants who had a higher BFM had an increased methylation level of *PGC-1α*. Due to its regulatory function in lipid and energy metabolism, it is likely that PGC-1α, in addition to its function in mitochondrial biogenesis, is also involved in metabolic diseases, such as obesity and diabetes [9]. A reduced *PGC-1α* expression could be observed in the skeletal muscle of patients with type 2 diabetes and in the adipose tissue of insulin-resistant and pathologically obese people [39]. It is possible that these are also found in the fatty tissue of healthy people with a slightly increased body fat percentage. This and the fact that the mRNA expression of *PGC-1α* correlates with the methylation highlight our results, even though our study did not include heavily overweight people.

Furthermore, we observed an association between *PGC-1α* methylation and the consumption of red and/or processed meat. Metabolic health as well as lifespan can be influenced by various nutrients, such as certain amino acids. Above all, the branched-chain amino acids (BCAAs) and methionine, which are particularly found in red meat, can regulate lifespan, aging, and metabolism through various mechanisms [40]. An excessive intake of red meat is also associated with age-related diseases such as cardiovascular diseases, type 2 diabetes, and cancer [41,42]. Animal studies as well as epidemiological studies have shown that reducing dietary BCAAs improves the Western diet–induced prevalence of obesity and glucose intolerance, and there is also an association between elevated BCAA levels and insulin resistance in obese and diabetic patients [40]. These elevated blood levels can trigger mitochondrial dysfunction. On the other hand, it should also be mentioned that supplementing BCAAs, especially in older people, can also have positive effects on health and mitochondrial biogenesis via mTOR, as shown in Figure 1 [40,43]. However, in addition to BCAAs, red or processed meat also contains other substances such as saturated fatty acids, heme iron, nitrosamines, sodium nitrite, L-carnitine, etc., which can have negative health consequences if consumed in excess [44]. These substances may be also responsible for the correlation between red meat consumption and *PGC-1α* methylation observed in this study. At minimum, there is evidence that the intake of the saturated fatty acids palmitic and stearic acid can impair mitochondrial function by reducing mitochondrial hyperpolarization and production of adenosine triphosphate (ATP). The treatment of cultured skeletal muscle cells with palmitic acid reduced *PGC-1α* expression [45]. However, clarifying data on the mechanisms in humans are still needed.

In addition to exercise, phytochemicals such as polyphenols in plant-based foods also seem to regulate the intracellular pathways of mitochondrial biogenesis [8]. We were able to point out an association between the consumption of cruciferous vegetables and mtDNA content. Cruciferous vegetables (e.g., broccoli, cabbage, or cauliflower) contain several nutrients, phytochemicals, and polyphenols such as carotenoids, chlorophyll, tocopherols, glucosinolates, flavonoids, sulforaphane, fibers, and a few others with health-promoting properties, such as potassium and folate [46,47]. The literature describes that polyphenols play a key role in replacing damaged mitochondria with newly synthesized mitochondria [48], and oxidative stress, e.g., induced by exercise, can modify mtDNA and lead to mitochondrial dysfunction. The consumption of cruciferous vegetables may maintain mtDNA levels via their antioxidant properties by fighting against oxidative stress and thereby affecting athletic performance [40,49]. Furthermore, mitochondria provide a significant portion of the energy of a muscle cell, and the more efficient the mitochondria are, the better an athlete’s performance [48]. In vivo and ex vivo studies have shown that isothiocyanate sulforaphane, from cruciferous vegetables, can activate NRF2 and increases the expression of *NRF1* and *TFAM* (Figure 1), supporting the model that sulforaphane influences mitochondrial biogenesis [50]. Although the effects of sulforaphane on mitochondrial biogenesis are largely uncharted, administration to rodents improved training performance and increased AMPK phosphorylation of skeletal muscles [50]. Our findings provide new evidence for the influence of sulforaphane on mitochondria via mtDNA content in humans, but further studies are needed to confirm study results, including a precise supplementation dose.

There are some potential limitations in this study that could be addressed in future research. Initially, this study focused on exercise- and diet-induced mitochondrial biomarkers; however, the functional aspects of mitochondria were not directly measured. This should be considered in future work. Second, unfortunately, no control group was studied, and the number of participants was relatively small. Nevertheless, we were able to identify exercise- and diet-related changes in both women and men using a minimally invasive method instead of muscle biopsy, which can be used for the general population and can be collected from anyone at any time.

## 5. Conclusions

In conclusion, our data support the model that concurrent exercise training affects different areas of athletic performance, e.g., mitochondrial biogenesis, and leads to positive effects on health. These are confirmed in particular by the decrease in body fat, increase in fat-free mass, and basal metabolic rate, which in turn leads us to expect better physical performance. In contrast to most previous scientific work using skeletal muscle biopsies, we measured the effects of exercise using a less invasive method (DBS) and epigenetic biomarkers such as miR-23a, miR-30e, and *PGC-1α* methylation, which may provide insight into exercise- and diet-induced mitochondrial biogenesis. Furthermore, our data showed that the consumption of red and/or processed meat has a negative impact on our measured biomarkers, whereas the intake of cruciferous vegetables has a positive effect. Taking this into account, eating habits may have an influence on athletic performance.

## Figures and Tables

**Table 1:** Training Plan. During strength training, there should be 60–120 s breaks between the sets. Training B would start the following Monday, followed by endurance training on Tuesday.

Monday Training A	Tuesday Endurance	Wednesday Training B	Thursday Break	Friday Training A	Saturday Endurance	Sunday Break
Warm up 5–10 min. stepper	30 to at least 60 min. in the last weeks of intervention	Warm up 5–10 min. stepper	-	Warm up 5–10 min. stepper	30 to at least 60 min. in the last weeks of intervention	-
Working sets Squats: 3 × 8–12 rep. Gluteus 3 × 8–12 rep. Chest press: 3 × 8–12 rep. Dumbbell row: 3 × 8–12 rep. Plank 3 × 8–12 rep.		Working sets Leg press: 3 × 8–12 rep. Lunges: 3 × 8–12 rep. Push-ups: 3 × 8–12 rep. Inverted row: 3 × 8–12 rep. Abdominal crunches 3 × 8–12 rep.		Working sets Squats: 3 × 8–12 rep. Gluteus 3 × 8–12 rep. Chest press: 3 × 8–12 rep. Dumbbell row: 3 × 8–12 rep. Plank 3 × 8–12 rep.		
Cool down 10 min stretching		Cool down 10 min stretching		Cool down 10 min stretching		

Rep = Repetition.

**Table 2:** Details of pyrosequencing assays and primers used to measure CpG methylation.

Assay ID	Primer	Sequence	No. of CpG Sites
*PGC1A*	fw:	5′-TAT AGT TAT TTT GTT ATG AAA TAG GGA GTT TTG -3′	1
	rev:	5′- biotin-CCA ATC ACA TAA CAA AAC TAT TAA AAA ATA A -3′	
	seq:	5′-GGA TTT TGG TTA TTA TAT GGT TAG G -3′	
	Sequence to analyze:	GTT TYG TTT AGA GTT TG	

Fw = forward, rev = reverse, seq = sequence, biotin = biotinylating.

**Table 3:** Participant characteristics.

	Total (*n* = 36)	Female (*n* = 21)	Male (*n* = 15)
Age ± SD [years]	31.86 ± 8.02	30.14 ± 8.51	34.27 ± 6.82
BMI ± SD [T0, kg/m^2^]	24.17 ± 2.95	23.78 ± 3.18	24.71 ± 2.61
BMI ± SD [T1, kg/m^2^]	23.88 ± 2.71	23.51 ± 2.95	24.40 ± 2.33
BMI classes			
Normal weight 18.5–24.9 kg/m^2^	58.3%	61.9%	53.3%
Overweight, 25–29.9 kg/m^2^	38.9%	33.3%	46.7%
Obesity grad I, 30–34.9 kg/m^2^	2.8%	4.8%	-
Basal metabolic rate ± SD [T0, kcal]	1481.11 ± 178.41	1355.71 ± 73.46	1656.67 ± 123.56
Basal metabolic rate ± SD [T1, kcal]	1495.81 ± 175.74	1369.05 ± 61.72	1673.27 ± 118.03
LBM ± SD [T0, kg]	51.93 ± 9.74	45.02 ± 4.96	61.60 ± 5.47
LBM ± SD [T1, kg]	52.44 ± 9.87	45.47 ± 4.72	62.20 ± 6.07
BFM ± SD [T0, kg]	20.06 ± 5.67	21.21 ± 6.08	18.44 ± 4.77
BFM ± SD [T1, kg]	18.53 ± 5.62	19.99 ± 5.84	16.47 ± 4.75
BFP ± SD [T0, %]	27.75 ± 6.21	31.24 ± 4.88	22.86 ± 4.31
BFP ± SD [T1, %]	26.04 ± 6.77	29.76 ± 5.27	20.83 ± 5.02
Intake frequencies			
Red/processed meat			
Rarely or never	44.4%	57.2%	26.6%
Once/week	25.0%	23.8%	26.6%
2–3×/week	22.2%	9.5%	40.0%
>4×/week or daily	8.4%	9.5%	6.8%
Cruciferous vegetables			
Rarely or never	58.3%	57.1%	60.0%
≥once/week	41.7%	42.9%	40.0%

SD = Standard Deviation, BMI = Body Mass Index, LBM = Lean Body Mass, BFM = Body Fat Mass, BFP = Body Fat Percentage.

**Table 4:** Main results of the analyzed marker.

Marker	Total (*n* = 36) 24–48 h Post-Exercise		Reduced Group (*n* = 27) 24–32 h Post-Exercise	
	Fold Change ± SD	*p* Value	Fold Change ± SD	*p* Value
*PGC-1α* Methylation	1.49 ± 1.42	0.826	1.88 ± 1.45	0.024 *
mtDNA	1.09 ± 0.37	0.426	1.10 ± 0.39	0.583
miR-23a-3p	0.92 ± 0.35	0.023 *	0.91 ± 0.36	0.028 *
miR-30e-3p	0.94 ± 0.34	0.047 *	0.94 ± 0.37	0.088

PGC-1α = Peroxisome Proliferator-activated Receptor Gamma Coactivator 1-Alpha, mtDNA = mitochondrial DNA, SD = Standard Deviation. * Shows significant *p*-values (paired *t*-test).

## Data Availability

Not applicable.
